# Neuroendoscopic Diagnosis and Treatment of Adolescent-Onset Aqueductal Stenosis: A Report of Two Cases Demonstrating the Utility of Endoscopic Third Ventriculostomy

**DOI:** 10.7759/cureus.96666

**Published:** 2025-11-12

**Authors:** Yuto Sakakibara, Mitsuharu Yamamoto, Takayuki Watanabe, Takayuki Ono, Koichiro Demura

**Affiliations:** 1 Neurosurgery, Toyokawa City Hospital, Toyokawa, JPN; 2 Neurosurgery, Nagoya City University East Medical Center, Nagoya, JPN

**Keywords:** adolescent hydrocephalus, endoscopic third ventriculostomy, late-onset aqueductal membranous occlusion, late-onset idiopathic aqueductal stenosis, neurocognitive outcome, shunt independence

## Abstract

Neuroendoscopic evaluation and endoscopic third ventriculostomy (ETV) are important in the management of adolescent-onset aqueductal stenosis, a condition often treated with a ventriculoperitoneal shunt (VPS). This condition is relatively rare, and treatment based on an accurate morphological assessment is desirable to provide less invasive and more precise therapy. We report two cases of adolescent-onset aqueductal stenosis (late-onset aqueductal membranous occlusion (LAMO) and late-onset idiopathic aqueductal stenosis (LIAS)) in which a definitive diagnosis was made by direct neuroendoscopic observation, leading to successful ETV and positive clinical outcomes. The clinical courses, imaging findings, intraoperative endoscopic findings, and postoperative courses of the two cases were retrospectively analyzed and compared with a review of the literature. Case 1 was a 16-year-old female patient with LAMO who presented with diplopia. Brain magnetic resonance imaging (MRI) suggested hydrocephalus and a membranous structure within the cerebral aqueduct. The preoperative Endoscopic Third Ventriculostomy Success Score (ETVSS) was 90. After confirming membranous occlusion endoscopically, ETV was performed. Her neurological symptoms completely resolved, and she was discharged with a modified Rankin scale (mRS) score of 0. No recurrence has been observed at 12 months after surgery. Case 2 was a 39-year-old male patient with LIAS who presented with a Glasgow Coma Scale score of 10. He had a history of VPS placement for aqueductal stenosis at age 17. A head computed tomography (CT) revealed acute hydrocephalus due to shunt malfunction. The preoperative ETVSS was 80. The aqueductal stenosis was confirmed endoscopically, diagnosed as LIAS, and ETV was performed. His consciousness improved, and he was transferred to another facility with an mRS score of 1. However, neurological deficits that developed during the acute hydrocephalic episode preceding ETV, such as a constricted visual field and cognitive dysfunction, persisted after surgery. No recurrence has been observed at three months after surgery. No procedure-related complications were observed in either case. In adolescent-onset aqueductal stenosis causing hydrocephalus, a treatment strategy of performing ETV after a definitive neuroendoscopic diagnosis appears safe and effective. This approach may avoid the lifelong risk of shunt-related complications and improve the patient's quality of life.

## Introduction

Among cases of non-communicating hydrocephalus, aqueductal stenosis is a primary cause. While the majority of cases are congenital and diagnosed in infancy, some cases do not manifest until adolescence [1]. Representative examples include late-onset idiopathic aqueductal stenosis (LIAS) and late-onset aqueductal membranous occlusion (LAMO) [1,2].

Conventionally, ventriculoperitoneal shunt (VPS) surgery has often been the standard treatment for such hydrocephalus. Although VPS remains an important treatment option, it is associated with complications such as infection, mechanical obstruction, over- or under-drainage, valve mismatch, and disconnection, any of which can lead to shunt malfunction [3]. The lifelong risk of shunt failure is considerably high, especially in young individuals, with 78.2% requiring revision surgery [3]. Furthermore, an increase in the number of shunt revisions may negatively impact a child's motor development and quality of life (QOL) [4]. For adolescent patients who are highly active and socially engaged, selecting a treatment method that accounts for these future medical, psychological, and economic burdens is critical.

In recent years, endoscopic third ventriculostomy (ETV) has been established as a minimally invasive, physiologic method of reconstructing cerebrospinal fluid (CSF) pathways for non-communicating hydrocephalus [1]. As ETV does not involve implanting a foreign body like a shunt system, it can, in principle, avoid the aforementioned risks. A recent systematic review has shown that ETV and VPS achieve comparable overall success rates, with pooled success of 81.8% for ETV and 86.7% for VPS at a median follow-up of 41 months; no significant difference in long-term success was detected between the two groups [5]. At the same time, postoperative complications were reported to be lower after ETV than after VPS [5]. Other studies suggest it may objectively improve a wide range of higher cognitive functions [1]. Against this backdrop, actively considering ETV, which can be a curative treatment, is critically important when considering the patient's QOL.

In this paper, we present two cases of adolescent-onset LAMO and LIAS. We discuss the validity of evidence-based treatment selection for ETV, the multifaceted evaluation of its clinical success, and the long-term significance of this treatment in young patients, supplemented by a review of the literature. In order to contextualize the expected prognosis of ETV in each case, we applied the Endoscopic Third Ventriculostomy Success Score (ETVSS). The ETVSS is a predictive index proposed by Kulkarni et al. that estimates the six-month success probability of ETV based on three factors: age, etiology of hydrocephalus, and prior shunt history [6]. The scoring system has been widely adopted in academic publications and is freely available for non-commercial scholarly use with appropriate citation. 

## Case presentation

Case 1: LAMO

**Figure 1:**
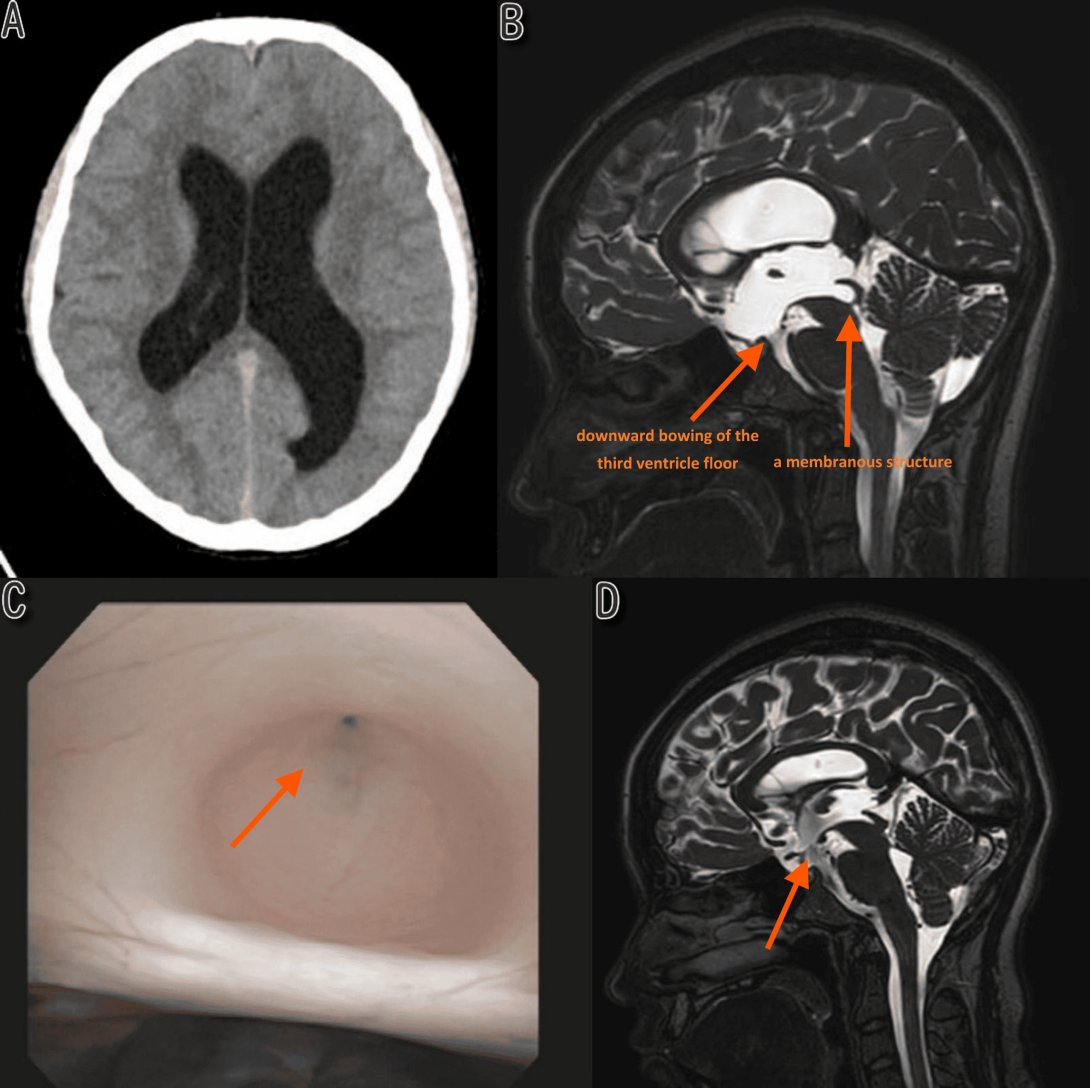
Case 1: (A) Preoperative CT. (B) Preoperative MRI T2WI. (C) Intraoperative endoscopy showing membranous occlusion. (D) Postoperative MRI at 12 months showing stoma patency CT: computed tomography; MRI: magnetic resonance imaging; T2WI: T2-weighted imaging

The patient was a 16-year-old female who presented with diplopia and a mild, persistent headache. Neurological examination revealed no abnormalities other than diplopia suggestive of abducens nerve palsy. A head computed tomography (CT) showed significant enlargement of the lateral and third ventricles (Figure 1A). On brain magnetic resonance imaging (MRI), a T2-weighted sagittal image revealed a low-signal linear shadow near the inlet of the cerebral aqueduct, suggesting occlusion by a membranous structure (Figure 1B).

Based on these findings, obstructive hydrocephalus, specifically LAMO, was suspected. The preoperative ETVSS was 90, calculated according to the method described by Kulkarni et al. [6], using age (≥10 years: 50 points), etiology (aqueductal stenosis: 30 points), and no prior shunt (10 points). Neuroendoscopic surgery was planned for both diagnosis and treatment. Under general anesthesia, a burr hole was made in the right frontal region, and a flexible neuroendoscope was inserted into the third ventricle via the foramen of Monro. After identifying the planned fenestration site in the tuber cinereum of the third ventricle floor, the aqueductal inlet was observed. A tense, translucent membranous tissue was found stretched across the inlet, obstructing the cerebral aqueduct (Figure 1C). Subsequently, the intermammillary recess of the third ventricle floor was perforated with forceps and dilated using a balloon catheter with a 4 mm tip to create an ETV stoma. We observed sufficient to-and-fro CSF pulsation through the stoma, indicating adequate restoration of outflow. We therefore did not proceed with aqueductal foraminoplasty in order to avoid the risk of hemorrhage or parenchymal injury in the periaqueductal and tectal regions. The surgery was completed after confirming no active bleeding.

The patient recovered well postoperatively. The diplopia completely resolved within 48 hours after surgery, and the patient was discharged with a modified Rankin scale (mRS) score of 0 [7]. At 12 months after surgery, MRI confirmed the patency of the ETV stoma (flow void sign positive) and sustained reduction in ventricle size (Figure 1D).

Case 2: LIAS

The patient was a 39-year-old male who had a history of VPS placement at age 17 at another hospital for hydrocephalus due to aqueductal stenosis of unknown etiology, which initially presented with visual impairment. After 22 years had passed since the surgery, he developed a headache and nausea, and within a few hours, he became drowsy with a Glasgow Coma Scale (GCS) score of 10 (E2V3M5) [8], resulting in emergency admission.

**Figure 2:**
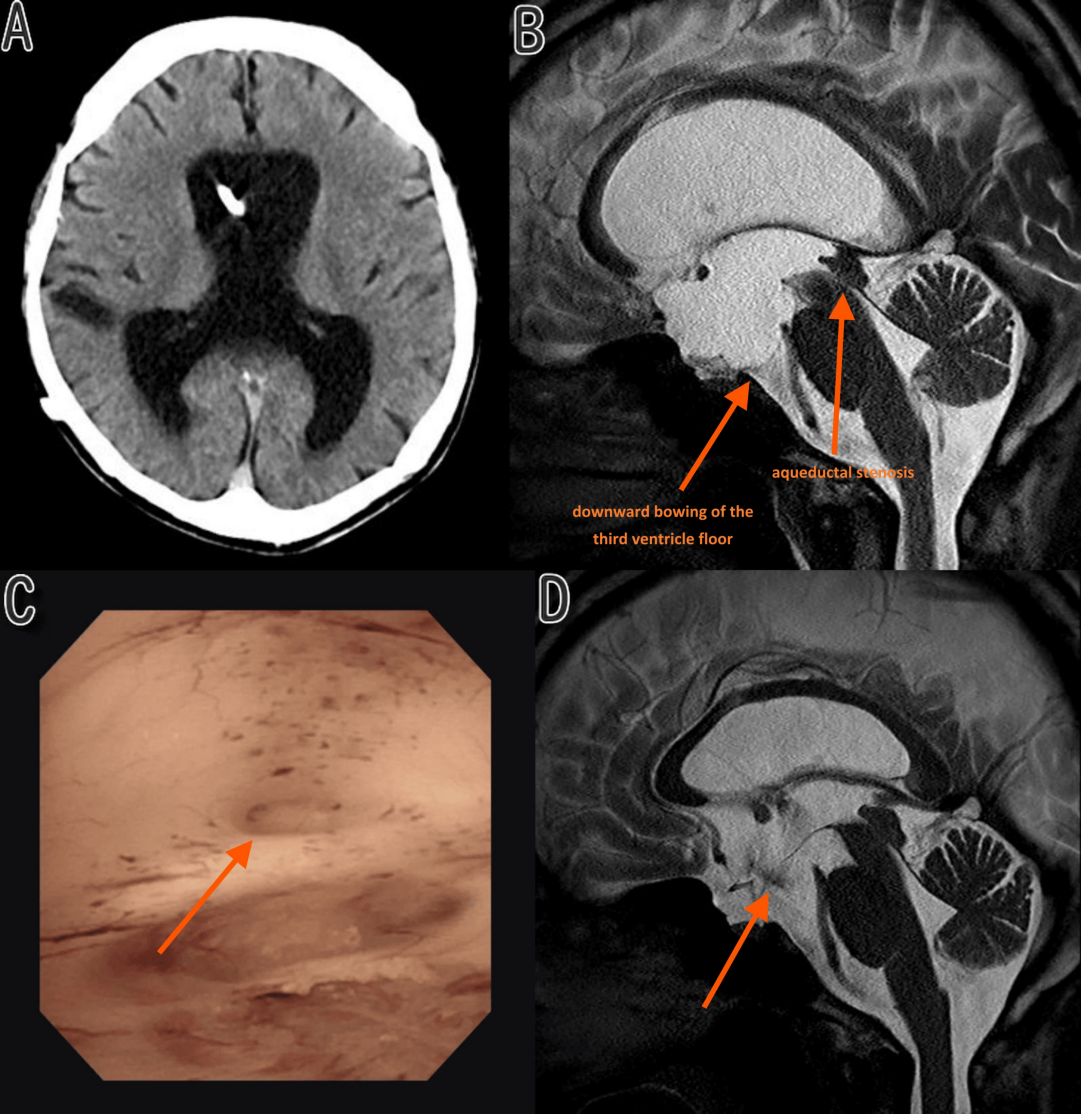
Case 2: (A) Preoperative CT (lateral ventricle enlargement due to shunt obstruction). (B) Preoperative MRI T2WI. (C) Intraoperative endoscopy showing scar stenosis. (D) Postoperative MRI on day 6 showing stoma patency CT: computed tomography; MRI: magnetic resonance imaging; T2WI: T2-weighted imaging

A head CT revealed enlargement of the lateral and third ventricles (Figure 2A). The programmable valve setting could not be adjusted, suggesting loss of proper valve function. CSF could still be aspirated from the prechamber, indicating patency of the proximal catheter, and a CT survey of the trunk demonstrated no fracture or disconnection of the distal catheter. Despite this, serial neuroimaging showed progressive ventricular enlargement. Taken together, these findings indicated acute hydrocephalus due to shunt malfunction. Brain MRI suggested aqueductal stenosis (Figure 2B). The preoperative ETVSS was 80, calculated according to Kulkarni et al. [6], using age (≥10 years: 50 points), etiology (aqueductal stenosis: 30 points), and prior shunt (zero points). Neuroendoscopic surgery was planned while preserving the existing VPS system. Under general anesthesia, a burr hole was made in the left frontal region, and a flexible neuroendoscope was inserted into the third ventricle via the foramen of Monro. Observation of the third ventricle floor and the aqueductal inlet revealed a white, scarred stenosis at the inlet, with no obvious membranous structure or tumorous lesion (Figure 2C), leading to a diagnosis of LIAS. The downwardly bulging and thinned tuber cinereum was perforated with forceps and dilated with a 4 mm tip balloon catheter to create an ETV stoma. Pulsatile bidirectional CSF flow was observed from the stoma. The surgery was completed after confirming no active bleeding.

The patient was endotracheally intubated immediately before surgery for the induction of general anesthesia. Because his level of consciousness remained depressed preoperatively, he was kept intubated overnight and was extubated the following day after a postoperative head CT confirmed no acute complications. His level of consciousness subsequently recovered to a GCS score of 15 [8] by the third postoperative day. Postoperative MRI on day 6 confirmed the patency of the ETV stoma (flow void sign positive) (Figure 2D). He was discharged to a rehabilitation facility on postoperative day 23 with an mRS score of 1 [7], reflecting persistent visual field deficits and subtle cognitive dysfunction but no major motor impairment. The existing shunt system was left in place since there were no signs of infection. Three months after surgery, he has had no recurrence of symptoms and has remained shunt-independent.

The clinical features and outcomes of the two cases are summarized in Table 1. Both patients have maintained shunt independence, and no recurrence of hydrocephalus has been observed.

**Table 1:** Clinical features and outcomes of the two cases LAMO: late-onset aqueductal membranous occlusion; LIAS: late-onset idiopathic aqueductal stenosis; GCS: Glasgow Coma Scale; VPS: ventriculoperitoneal shunt; ETVSS: Endoscopic Third Ventriculostomy Success Score; mRS: modified Rankin scale

Characteristics	Case 1 (LAMO)	Case 2 (LIAS)
Age/sex	16-year-old female	39-year-old male
Chief complaint	Diplopia, headache	Acute headache, nausea, decreased level of consciousness (GCS=10)
Past medical history	None	VPS placement at age 17 (functional for 22 years)
Pathophysiological mechanism	Membranous occlusion	Intrinsic stenosis
Head circumference	Within normal range	Within normal range
Key preoperative imaging findings	Membranous structure in the aqueduct; third and lateral ventricle enlargement	Aqueductal stenosis; significant enlargement of the third and lateral ventricles
ETVSS	90	80
Endoscopic diagnosis	LAMO (translucent membranous tissue at the aqueductal inlet)	LIAS (stenosis at the aqueductal inlet, no membranous structure)
Surgical complications	None	None
Follow-up period	12 months	3 months
Neurological outcome	Complete resolution of symptoms	Marked improvement of symptoms
mRS (at the final evaluation)	0	1
Final status	Shunt-independent	Shunt-independent

## Discussion

Significance of endoscopic observation in morphological diagnosis

MRI is essential in the initial evaluation of aqueductal stenosis, but it may yield false negatives if the membranous structure in LAMO is extremely thin. In Case 1, although preoperative MRI suggested the presence of a membranous structure, MRI alone could not fully characterize the aqueductal inlet, particularly with respect to thin membranous obstruction at the entrance of the aqueduct. Neuroendoscopy allowed direct, detailed visualization of the proximal aqueduct and surrounding anatomy. This enabled us to distinguish a thin membranous occlusion consistent with LAMO from intrinsic aqueductal stenosis consistent with LIAS. Importantly, neuroendoscopy is not used solely as a confirmatory diagnostic tool; real-time endoscopic inspection directly informs intraoperative management by indicating whether ETV alone is sufficient or whether additional maneuvers such as endoscopic aqueductoplasty (EAP) or stent placement are warranted. Nakamura et al. reported that EAP is often performed in cases of LAMO with good outcomes [9]. On the other hand, some reports indicate that EAP has a considerably high long-term re-occlusion rate of 88.2% and should be considered only if a stent is placed concurrently [10]. Therefore, endoscopic morphological diagnosis is considered to play a crucial role in determining the treatment plan. In our cases, both were manageable with ETV alone, but in some instances, endoscopy can serve not only as a diagnostic tool but also as a means for immediate therapeutic intervention.

Treatment outcomes of ETV and challenges of VPS

The success rate of ETV for LIAS is reported to be over 80% in many studies [11]. For LAMO, although case reports are few, either ETV, EAP, or both have been performed, with many reports showing positive clinical outcomes [9]. The long-term results of ETV for pediatric aqueductal stenosis are also good, with a five-year revision-free survival rate of 89%. However, since late stoma closure leading to recurrence can occur, albeit rarely, cautious long-term follow-up is important [12]. In our series, both adolescents achieved durable control without recurrent symptomatic hydrocephalus: Case 1 remained clinically stable and shunt-independent at 12 months of follow-up, and Case 2 remained stable without recurrent ventriculomegaly at three months of follow-up.

In contrast, while VPS remains an important treatment option for hydrocephalus, it is associated with numerous long-term problems, especially in younger patients. According to Reddy et al., because shunted patients face lifelong risks such as mechanical obstruction, infection, valve pressure mismatch, overdrainage, and disconnection, the overall shunt success rate was 53.7% [3]. In that cohort, 32.5% of adults and 78.2% of children ultimately required shunt revision, with most revisions occurring within the first six months after the index surgery [3]. It has also been reported that an average of 2.7 revisions is required [3]. These revisions not only significantly decrease the patient's QOL but have also been suggested to affect neurodevelopment [4].

The ETVSS proposed by Kulkarni et al. predicts six-month ETV success [6]. An ETVSS ≥70 indicates that ETV's success rate is comparable to or better than VPS, and a score ≥80 suggests ETV is clearly superior in the early postoperative period, suggesting that ETV is advantageous not only in its long-term benefits but also in its short-term safety. However, it must be noted that this score was developed based on a pediatric cohort. Subsequent validation studies in mixed populations, including adults, have pointed out that while the score is good at predicting success rates for the group, its ability to discriminate success or failure for individual patients is insufficient [13]. Therefore, in adult cases, radiological factors not included in the ETVSS, such as downward bowing of the third ventricle floor, should be considered [14]. In our report, Case 1 had an ETVSS of 90 and Case 2 had a score of 80, calculated according to Kulkarni et al. [6]. Especially for Case 2, an adult patient, we also emphasized findings like the downward bowing of the third ventricle floor and anticipated a high likelihood of ETV success in both cases. The reported success rate for secondary ETV is 63%, which is relatively modest [15]. Nevertheless, a comprehensive judgment considering factors such as adolescent-onset aqueductal stenosis and the aforementioned radiological findings suggests that achieving shunt independence, even after a long period of shunt dependency as in Case 2, is possible.

Characteristics and treatment strategy of adolescent-onset aqueductal stenosis

An important differential diagnosis for adolescent-onset aqueductal stenosis is long-standing overt ventriculomegaly in adults (LOVA). LOVA is said to present with characteristic imaging findings such as marked ventriculomegaly, macrocephaly, and destruction of the sella turcica [16]. In contrast, LIAS patients typically have a normal head circumference and a higher prevalence of gait disturbance, urinary incontinence, and dementia, in addition to symptoms of increased intracranial pressure such as headache [16]. Our cases did not exhibit macrocephaly and presented with clinical features suggesting increased intracranial pressure, which was considered different from LOVA. Moreover, ETV alone may not lead to complete symptom resolution in LOVA [17]. Therefore, distinguishing LOVA from adolescent-onset aqueductal stenosis is crucial for determining the optimal treatment.

Adolescence is a critical period for personality development. In treating hydrocephalus during this period, not only symptom improvement but also the maintenance and enhancement of neurocognitive function are important treatment goals. Although we did not perform objective neuropsychological testing in our cases, studies show that ETV for LIAS can objectively improve a wide range of higher brain functions, including attention, executive function, visuospatial memory, verbal executive function, and even behavioral and emotional domains [1]. On the other hand, VPS for non-infectious hydrocephalus in children may have adverse effects on mental and motor development [18]. Thus, ETV is considered to contribute significantly to improving the QOL of adolescent patients. Furthermore, avoiding the implantation of a foreign body like a shunt device is also important, as it bypasses cosmetic concerns, worries about activity restrictions, and the psychological burden of anxiety over malfunction.

Long-term perspective

As mentioned above, VPS is associated with various complications and the risk of sudden malfunction and emergency surgery. In contrast, although the follow-up period in our cases is short, many studies have shown that ETV can be expected to have long-term durability [12]. In a long-term follow-up study of adults over 10 years, the success rate of ETV was high at 73%, with the majority of failures occurring within the first two years after surgery [19]. However, as the risk of late-onset obstruction is not nonexistent, it is essential to educate patients about the symptoms of recurrence (symptoms of increased intracranial pressure) and to establish a system for careful lifelong follow-up.

This avoidance of shunt dependency brings significant benefits not only in improving the patient's QOL but also in terms of healthcare economics. The costs associated with treating shunt-related complications and revision surgeries are considerable. Therefore, when considering lifetime costs, it is suggested that ETV is far more cost-effective than VPS, even if the initial costs are comparable or higher [20]. For young patients with a long life expectancy, ETV is not merely a symptomatic treatment but a curative intervention that can alter the natural history of a chronic disease and enable them to lead a higher QOL.

Limitations of this study

This study is a retrospective, single-center report of two cases with a short follow-up period. Accordingly, the findings should be regarded as hypothesis-generating rather than definitive. Additionally, because standardized pre- and postoperative neuropsychological testing was not performed, objectively demonstrating cognitive or QOL change is difficult in our study. Therefore, any discussion of neurocognitive benefit is presented as a possibility informed by prior reports rather than as a measured effect in these cases. To establish the optimal treatment for adolescent-onset aqueductal stenosis, multicenter prospective studies and analyses of larger case series with longer follow-up are necessary.

## Conclusions

We report two adolescent-onset aqueductal stenosis cases (one LAMO and one LIAS) treated with ETV after neuroendoscopic confirmation of the diagnosis, both with successful outcomes. A diagnostic approach using a neuroendoscope ensures accurate differentiation between these pathologies and enables the selection of the optimal treatment method. For adolescent patients with aqueductal stenosis who have good prognostic indicators, a treatment strategy with ETV as the first choice is considered a safe, effective, and important treatment option that can avoid lifelong shunt dependency and its associated complications.
